# Factors Associated With Falls Among Urban-Dwellers Aged 55 Years and Over in the Malaysian Elders Longitudinal Research (MELoR) Study

**DOI:** 10.3389/fpubh.2020.506238

**Published:** 2020-11-16

**Authors:** Deepa Alex, Hui M. Khor, Ai V. Chin, Noran N. Hairi, Robert G. Cumming, Sajaratulnisah Othman, Selina Khoo, Shahrul B. Kamaruzzaman, Maw P. Tan

**Affiliations:** ^1^Jeffrey Cheah School of Medicine and Health Sciences, Monash University Malaysia, Subang Jaya, Malaysia; ^2^Division of Geriatric Medicine, Department of Medicine, University of Malaya Medical Centre, Kuala Lumpur, Malaysia; ^3^Ageing and Age-Associated Disorders Research Group, Faculty of Medicine, University of Malaya, Kuala Lumpur, Malaysia; ^4^Department of Social and Preventive Medicine, Faculty of Medicine, Julius Centre, University of Malaya, Kuala Lumpur, Malaysia; ^5^School of Public Health, University of Sydney, Sydney, NSW, Australia; ^6^Department of Primary Care Medicine, Faculty of Medicine, University of Malaya, Kuala Lumpur, Malaysia; ^7^Centre for Sport and Exercise Sciences, University of Malaya, Kuala Lumpur, Malaysia; ^8^Department of Medical Sciences, School of Healthcare and Medical Sciences, Sunway University, Kuala Lumpur, Malaysia

**Keywords:** falls, risk factor, older population, Asia, incontinence

## Abstract

Falls are major issues affecting the older population with potentially serious complications, including fractures, head injury, institutionalization, fear of falling and depression. While risk factors for falls have been established across Western Europe and North America, geographical differences in falls risk have not been well researched. We aim to examine the clinical and physical risk factors for falls in a middle-income South East Asian country. Cross-sectional data from the Malaysian Elders Longitudinal Research (MELoR) study involving 1,362 community dwelling individuals aged 55 years and above was utilized. Information on sociodemographic and medical history was obtained by computer-assisted questionnaires completed during home visits and hospital-based detailed health checks. Univariate and multivariate analyses compared non-fallers and fallers in the previous 12 months. Urinary incontinence, hearing impairment, depression, arthritis and cognitive impairment were risk factors for falls in the past 12 months after adjustment for age in our study population. Awareness about the risk factors in a population helps the design of fall prevention strategies that target specific or multiple risk factors.

## Introduction

One in four adults aged 65 years and over fall at least once in 12 months (1–3). Falls are associated with fractures, head injury, and other potentially life-threatening injuries (4). In addition, after a fall, the older person may develop fear of falling and other psychological consequences, such as depression (5, 6). Falls also lead to serious social consequences such as institutionalization and increased demands for social care (7). Several cost economic analyses have demonstrated high economic costs associated with falls (8).

Numerous risk factors for falls have been published over the past five decades. The most commonly reported risk factors include muscle weakness, visual impairment, environmental hazards, cardiovascular disease and medications (9). As a result, numerous studies have been published to support the effects of exercise intervention as a primary and secondary prevention measure (10). Multi-factorial interventions which target two or more risk factors simultaneously have also been shown to reduce frequency of falls as a secondary prevention measure (11).

Existing studies on falls risk factors have primarily been conducted in developed nations (12). With the rapidly increasing proportion of the older population in South East Asia, falls is becoming an issue of increasing concern in this region. Limited studies currently exist for falls risk factors for South East Asia (SEA). Evidence from other SEA studies suggest that some risk factors for falls are unique to the region due to local and cultural factors (13). We have therefore conducted a cross sectional analysis of the first wave data from the Malaysian Elders Longitudinal Research (MELoR) study to identify clinical and physical risk factors associated with falls in a middle-income Asian community. The rationale for using a cut-off of 55 years was that the mandatory retirement age for Malaysia at the time of study commencement was 55 years, and this enabled the analysis of health issues of individuals as they approached retirement.

## Methods

The Malaysian Elders Longitudinal Research (MELoR) study is an interdisciplinary research initiative evaluating issues associated with aging within the older communities surrounding the University Malaya. Participants for the study were individuals aged 55 years and above who were residents of the three parliamentary constituencies of Pantai Valley, Petaling Jaya North and Petaling Jaya South which are urban areas in greater Kuala Lumpur. Potential participants were selected through simple random sampling stratified by age deciles and the three main ethnic groups of Malay, Chinese and Indian ethnicities. This cross-sectional study included data from 1,362 participants from the MELoR study. A detailed description of the cohort study can be accessed elsewhere (14). This study has been approved by the University of Malaya Medical Center Ethics Committee (Ref: 925.4).

### Data Collection

Participants were contacted and visited at their own homes initially to recruit them into the study. Written informed consent was obtained from all participants. Trained researchers administered the questionnaire during these home visits that occurred between October 2013 to May 2015. A structured interview using a computer aided questionnaire was completed during this encounter. Information on sociodemographic and medical factors were collected as well as falls history. Participants were then requested to attend the hospital for a detailed health check. Physical and cognitive measurements were recorded during the health check.

### Falls

The presence of a fall during the 12-month period preceding the date of the interview was established using the question, “Have you fallen in the past 12 months?” A fall was defined as unintentionally coming to rest on the ground, floor, or lower level (15).

### Independent Variables

Potential risk factors for falls were selected based on previous published studies (16, 17). The sociodemographic factors taken into account were age (18), gender (19), living alone (20), educational level (21), marital status (20), and smoking status (19). Medical factors included number of comorbidities (22), number of prescribed medications (22), presence or absence of self-reported medical conditions such as vision impairment (23), hearing impairment (24), urinary incontinence (25), Parkinson's disease (26), stroke (27), transient ischemic attack, heart disease, arthritis, depression (28), diabetes mellitus (29), chronic kidney disease (28), chronic obstructive pulmonary disease (30), malignancy (31), and cognitive impairment (32). The number of comorbidities was calculated from the total number of 14 self-reported medical conditions.

### Urinary Incontinence

The presence of urinary incontinence was identified during the home-based, computer-assisted interview, by inquiring about symptoms of stress and urge incontinence through the questions “Do you ever wet yourself when you cough or strain?” and “Do you ever wet yourself before you reach the toilet?” respectively. Participants who admitted to the presence of either or both symptoms were classified as having urinary incontinence.

### Cognitive Impairment

Cognitive impairment was assessed using the Montreal Cognitive Assessment (MoCA) (33). The MoCA was administered during the hospital-based health check using participants' preferred language including English (34), Bahasa Malaysia (35), Singaporean Mandarin Chinese (34), and Tamil (34). The test was administered by trained researchers fluent in each language, under standardized conditions. The MoCA measures cognitive performance in the domains of attention and concentration, executive function, memory, language, visuoconstructional skills, conceptual thinking, calculation, and orientation. The overall score is obtained by the total of each domain score. The presence of cognitive impairment is defined as a MoCA score below 26 out of a potential maximum score of 30.

### Physical Performance Measures

Low physical performance measures have been shown to be associated with high risk of falls in older adults (36). Physical factors assessed in this study included grip strength (37), the timed up and go test (TUG) (38), walking speed (39), and functional reach (40).

#### Handgrip Strength

Handgrip strength (HGS) was measured using a calibrated electronic Jamar grip strength dynamometer (Jamar Plus+, Sammons Preston USA). Participants were instructed to grip as hard as they could while seated upright on a chair, with their elbow flexed at 90° and their arm adducted. The average of three readings obtained from the dominant hand was considered in this study.

#### Timed-Up-and-Go

Gait and balance were assessed using the timed-up-and-go test (38). The total time taken for the participant to rise from a seated position from a standard chair with arms, stand up, walk to a mark 3 m away, turn around and return to the chair was measured in seconds. Individuals who took 13.5 or more seconds to perform the test were considered at increased risk of falls, and included in the impaired TUG group (41).

#### Walking Speed

Walking speed was measured over 15 feet with a flying start. The stopwatch was started as soon as the big toe of the participant's leading foot crossed the starting line and stopped when the participant's leading big toe crossed the 15-foot marker. Reduced walking speed was defined as a 15-foot walk of 6 s or above for male participants with a standing height of >173 cm and for female participants with a standing height >159 cm, and ≥7 s for men with a height of 173 cm and below and women with a height of 159 cm and below (39).

As the TUG test assessed a person's mobility while walking speed was indicative of slowness, both physical markers of frailty, a new variable of impaired TUG “OR” impaired walking speed was created.

#### Functional Reach

Functional reach (FR) is a test to assess dynamic balance. The maximal distance one can reach forward beyond arm's length, while maintaining a fixed base of support in the standing position is the Functional reach. This was achieved by attaching a meter rule to the wall at shoulder height, parallel to the floor. Participants were instructed to stand with their left shoulder next to the wall, but not touching the wall, with the body perpendicular to the wall. The initial position of the first interphalangeal joint of the middle finger with the participant standing upright was recorded. The subsequent position of the first interphalangeal joint of the middle finger with the participant at maximal forward reach, with the arm outstretched and maintained in a position next to the meter rule was recorded (42).

### Statistical Analysis

Categorical variables were presented as frequencies with percentages in parentheses. The presence and absence of falls were compared for each variable using the chi-squared test for categorical variables. The continuous variables (number of comorbidities, grip strength and functional reach) were divided into quartiles and evaluated using logistic regression with dummy variables. The variable number of medications was divided into tertiles, while TUG and 15-feet walking time were combined and dichotomized into those with and without abnormal TUG or walking speed. For factors with a positive association with falls, the lowest groups (e.g., number of comorbidities = 0, number of medications ≤1) were considered the reference group, while for factors with a negative association with falls, the highest group (e.g., grip strength >39 kg, functional reach >37.5 cm) were considered the reference category. A logistic regression analysis was conducted to determine the odds ratio (OR) with 95% confidence interval (CI) for each variable and variables with a *p*-value of < 0.2 were initially selected for multivariate analysis in order to identify a predictor model for risk of having fallen at least once in 12 months based on our population data. A *p*-value of < 0.05 was taken as statistically significant.

## Results

Health check, physical performance and cognitive assessment data were available for 1,362 individuals. Of these, 312 (22.9%) sustained at least one fall in the preceding 12 months. The basic characteristics of the overall study population is shown in Table 1. Fallers were significantly older, more likely to be female, more likely to be of “single/widowed/separated” marital status and were more likely to have lower educational attainment.

**Table 1:** Sociodemographic characteristics of fallers vs. non-fallers.

**Variable**	**Fallers**	**Non-fallers**	***p*-value**
Age categories^*^			
55–59 years	29 (9.3)	144 (13.7)	0.001
60–64 years	61 (19.6)	224 (21.4)	
65–69 years	71 (22.8)	255 (24.3)	
70–74 years	66 (21.2)	254 (24.2)	
≥75 years	84 (27.0)	171 (16.3	
Females^*^	199 (64.2)	578 (55)	0.005
Primary education^*^	95 (30.6)	260 (24.8)	0.047
Single/widowed/separated^*^	93 (30)	246 (23.5)	0.025
Living alone^*^	16 (5.4)	53 (5.3)	0.883
Smokers^*^	52 (17.3)	208 (20.2)	0.321

* *Frequency (Percentage)/p-value derived by chi square*.

### Univariate Analysis

The clinical risk factors which were significantly more likely to be present among fallers after adjusting for age were: Parkinson's disease, diabetes mellitus, arthritis, depression, visual impairment, hearing impairment, cognitive impairment, and transient ischemic attack (TIA) (Table 2). Fallers were also more likely to consume five or more prescribed medications. The presence of two or more comorbidities was associated with falls compared to absence of comorbidities, with the odds ratio increasing with number of comorbidities. The presence of stress or urge incontinence was also significantly associated with falls.

**Table 2:** Univariate and multivariate analysis for predictors of falls in the preceding 12 months.

**No**.	**Characteristic**	**Unadjusted**		**Adjusted for age**		Adjusted for all covariates^**^	
	**Medical risk factors**	***p*-value**	**OR (95% CI)**	***p*-value**	**OR (95% CI)**	***p*-value**	**OR (95% CI)**
1.	Number of comorbidities 0 (Reference)						
	1	0.108	1.55 (0.91–2.65)	−0.153	1.48 (0.86–2.53)	0.777	0.90 (0.44–1.82)
	2	0.002	2.29 (1.36–3.85)	0.004	2.17 (1.29–3.66)	0.370	0.68 (0.29–1.58)
	3 or more	<0.001	3.99 (2.40–6.63)	<0.001	3.65 (2.18–6.10)	0.273	0.52 (0.16–1.66)
2.	Vision impairment	0.024	1.87 (1.08–3.24)	0.019	1.03 (1.01–1.05)	0.295	1.54 (0.68–3.46)
3.	Hearing impairment	<0.001	1.68 (1.26–2.24)	0.002	1.59 (1.19–2.12)	*0.002*	*2.00 (1.28*–*3.12)*
4.	Urinary incontinence	<0.001	1.79 (1.36–2.37)	<0.001	1.78 (1.35–2.35)	*0.015*	*1.74 (1.11*–*2.73)*
5.	Stroke	0.11	2.17 (0.83–5.66)	0.15	2.02 (0.77–5.28)	0.54	1.48 (0.42–5.19)
6.	Transient ischemic attack	0.03	1.99 (1.06–3.73)	0.05	1.85 (0.98–3.48)	0.20	1.68 (0.75–3.75)
7.	Parkinson's disease	0.012	5.13 (1.44–18.32)	0.016	4.82 (1.34–17.30)	0.52	1.83 (0.27–12.13)
8.	Heart disease	0.48	0.86 (0.57–1.29)	0.27	0.79 (0.52–1.19)	–	–
9.	Chronic obstructive pulmonary disease	0.58	1.45 (0.37–5.66)	0.73	1.27 (0.32–5.02)	–	–
10.	Chronic kidney disease	0.06	1.84 (0.97–3.48)	0.07	1.78 (0.94–3.39)	0.745	0.87 (0.37–2.03)
11.	Diabetes mellitus	0.001	1.57 (1.20–2.06)	0.002	1.51 (1.15–1.99)	0.060	1.55 (0.98–2.45)
12.	Arthritis	<0.001	2.05 (1.50–2.79)	<0.001	1.97 (1.44–2.69)	*0.004*	*2.03 (1.25*–*3.28)*
13.	Depression	0.001	4.25 (1.75–10.36)	0.002	4.16 (1.70–10.21)	*0.017*	*4.51 (1.30*–*15.60)*
14.	Malignancy	0.946	1.01 (0.60–1.70)	0.847	0.95 (0.56–1.61)		–
15.	Cognitive impairment	<0.001	1.66 (1.25–2.20)	0.001	1.59 (1.19–2.11)	*0.031*	*1.68 (1.05*–*2.69)*
16.	Number of medications≤ 1 (Reference)						
17.	2–4	0.142	1.24 (0.93–1.64)	0.378	1.14 (0.85–1.53)	0.840	0.96 (0.67–1.38)
18.	≥5	0.001	2.87 (1.53–5.40)	0.007	2.41 (1.27–4.60)	0.210	1.67 (0.74–3.77)
	**Physical risk factors**						
19.	TUG/ 15 feet walking speed	<0.001	1.83 (1.41–2.38)	<0.001	1.71 (1.30–2.25)	0.096	1.32 (0.95–1.84)
20.	Handgrip strength >39 kg (Reference)						
	≤ 13 kg	0.002	5.76 (1.93–17.19)	0.004	4.93 (1.64–17.79)	0.072	4.14 (0.88–19.43)
	13.1–26 kg	0.006	4.29 (1.53–12.03)	0.008	4.02 (1.43–11.28)	0.030	4.98 (1.16–21.35)
	26.1–39 kg	0.127	2.28 (0.79–6.57)	0.147	2.19 (0.76–6.32)	0.123	3.18 (0.73–13.83)
19.	Functional reach >37.5 cm (Reference)						
	≤ 12.5 cm	0.001	5.85 (2.05–16.69)	0.003	5.08 (1.76–14.64)	0.207	2.13 (0.65–6.90)
	12.6–25 cm	0.011	3.36 (1.31–8.59)	0.020	3.04 (1.18–7.82)	0.899	1.06 (0.38–2.94)
	25.1–37.5 cm	0.076	2.34 (0.91–6.00)	0.100	2.20 (0.86–5.66)	0.942	0.96 (0.35–2.61)

** *Variable(s) entered: Age, Hearing, Vision, Incontinence, Stroke, TIA, Parkinson's Disease, Diabetes, Chronic Kidney Disease, Arthritis, Depression, Cognitive impairment, Number of medications, Grip strength categories, Functional reach categories, Impaired TUG/15 feet walking speed, Number of comorbidities. p-values < 0.05 in the final model under the column “adjusted for all covarites” are in italics*.

Individuals with a grip strength of 13 kg or less, and 13.1–26 kg, were at increased risk of falls compared to individuals with grip strength of above 39 kg, but not those with a grip strength of 26.1–39 kg. Similarly, individuals in the functional reach groups of 12.5 cm or less and 12.6–25 cm were associated with greater risk of falls compared to those with functional reach of 37.6 cm or greater. However, those with functional reach of 25.1–37.5 cm were not significantly associated with a higher risk of falls, when compared to those with functional reach of 37.6 cm or greater. Therefore, lower grip strength and functional reach were associated with a higher risk of falls. The Impaired TUG or 15-feet walking speed were associated with increased risk of falls.

### Predictor Model for Falls Risk

The final model for the multivariate analysis revealed that incontinence, hearing impairment, depression, arthritis and cognitive impairment were independently associated factors for falls after adjustment for all covariates (Table 2).

## Discussion

Few studies have evaluated risk factors for falls in older community-dwelling populations in Malaysia as well as South East Asia. Our study revealed that presence of urinary incontinence, hearing impairment, depression, arthritis and cognitive impairment were significant risk factors for falls in our population.

The relationship between urinary incontinence and falls has been brought to prominence only in the last decade. Urinary incontinence and falls, both being the most common geriatric giants in community living older persons (43), are associated with functional decline and disability. There is a complex interplay of mechanisms which link urinary incontinence and falls which eventually leads to disability (44). Symptoms of urge incontinence such as rushing to the toilet to avoid social embarrassment could potentially trigger a fall (45). Increased frequency of micturition magnifies this risk. Presence of nocturia may cause daytime drowsiness which may lead to increased risk of falls (45). Use of diuretics which is associated with frequency, nocturia and incontinence may also contribute to an increased risk of falls, through additional hemodynamic adverse effects of postural hypotension which has not been adjusted for in this study (46). It has been noted in previous studies that urinary incontinence by itself, as well as stress and urge incontinence are individually associated with a higher risk of falling (47). This is similar to our study, whereby the odds of sustaining a fall were 1.7 times higher in the presence of stress or urge incontinence or both.

Hearing impairment is a highly prevalent yet under-treated issue among older adults. Previous studies have shown that hearing loss is a risk factor for falls, possibly explained by concomitant cochlear and vestibular dysfunction, or lack of awareness of the spatial environment (24). Our study has also identified self–reported hearing impairment as an independent risk factor for falls. The mechanism underlying hearing difficulties and falls is not yet well understood. Neither is there clarity of whether correcting hearing deficits using available hearing augmentation devices will reduce falls risk.

Arthritis was found to be significantly associated with falls in our study population. Similar findings were noted in community dwelling older adults in the US (48) as well as in the British Women's Heart and Health Study (49). The link between arthritis and falls can be explained by various factors such as joint pain leading to an increased risk of falls (50), decreased function due to stiffness and poor mobility, and usage of opioid analgesics that contribute to falls through dizziness (51). However, in this study the information on the type of arthritis, details of number of joints affected, severity and radiographic evidence of arthritis was lacking. Moreover, the definition of arthritis was based on self-report by the participant.

Although depression is an established risk factor for falls in existing literature (52), the mechanism underlying the link between the two entities is still unclear. There are various postulated mechanisms proposed to explain this association. Gait and balance can be affected in depression through impaired concentration (53) and impaired neurological reflexes leading to postural abnormalities (54). The association between late life depression and falls could also be explained by cardiovascular autonomic abnormalities (55). The use of antidepressants can lead to an increased falls risk through impaired gait, balance and blood pressure regulation (56). Social factors can play an important role as social isolation in depressed individuals can lead to decreased physical activity which then leads to muscle weakness, thereby increasing the risk of falls (57). This study has revealed that depression is independently associated with falls in the MELoR study population but further research will be needed to elucidate the mechanisms underlying this association.

Cognitive impairment is an established risk factor for falls (58). Specific cognitive domains such as planning and working memory have been linked to gait and fall risk (59). Impairment in attention and executive function have been found to linked with gait variability and falls (60). Slower reaction times could lead to delayed postural response thereby increasing the likelihood of falls (61). Fall prevention strategies to date are targeted toward cognitively intact older adults and they may not be as successful in decreasing falls risk in persons with cognitive impairment. As cognitive impairment has been identified as an independent risk factor for falls in our study, future fall interventions need to be designed, keeping in mind the needs of individuals with cognitive impairment as they may need specifically tailored therapies for effective fall prevention.

Despite polypharmacy being previously considered an establish falls risk factor, this did not emerge as an independent associated risk factor in this study. This is likely to be attributed to the confounding effects of multiple comorbidities. Previous studies have highlighted the difficulty in adjusting for the presence of individual medical conditions when evaluating the relationship between medications and falls (62). Recent authors have suggested that falls risk increasing drugs and inappropriate prescribing should replace polypharmacy as the target for medication review (63).

As this was a cross-sectional study, we are unable to assign causation for our risk factors. The medical conditions taken into consideration as risk factors for falls were self-reported by the participants of the study. The presence of falls in the preceding year was reported by retrospective recall, so there could have been possible under-reporting of falls. The study was conducted in an urban population, so it may not be representative of the entire Malaysian older population. However, 70% of all Malaysians now live in urban areas. The predictor model also reflected prediction of the presence of falls in the preceding 12 months, rather than prospectively, and may hence have limited utility. However, this is nevertheless an important finding given the sparse data available for the region, and will go on to inform future prospective studies to confirm these findings. In addition, the presence of available risk factors in a busy clinic setting may be used as electronic prompts to attending physicians to inquire about previous falls, which in turn is considered an important predictor of future falls, with previous studies indicating that the presence of a previous fall is associated with doubling in the risk of future falls (64).

A round table report from seven Asian countries on fall prevention strategies noted that recommendations specific to the Asian population are necessary for effective fall prevention programs in the region (65). The most common approaches around fall prevention in the region were exercise interventions, educational programmes or a combination of both. Our study has highlighted that future interventions should also consider targeting specific issues such as incontinence and hearing impairment in community dwelling older adults. Fall prevention strategies need to be designed that are effective for older adults with cognitive impairment and depression which were identified as independent risk factors for falls in our study population.

## Conclusion

The predictive risk factors for the presence of at least one fall in the preceding 12 months in an urban community-dwelling population aged 55 years and over in Malaysia were urinary incontinence, hearing impairment, depression, arthritis and cognitive impairment after adjustment for age. While our findings need further confirmation through prospective studies, effective strategies for fall prevention should also consider focusing on early detection and management of the above risk factors.

## Data Availability Statement

The datasets generated for this study are available on request to the corresponding author.

## Ethics Statement

The studies involving human participants were reviewed and approved by University of Malaya Medical Center Ethics Committee (Ref: 925.4). The patients/participants provided their written informed consent to participate in this study.

## Author Contributions

AC, NH, SO, SK, SBK, HK, and MT conceived the study, contributed to study design, obtained the funding for the study, and were responsible to the conduct of the study. DA, HK, and MT contributed to data analysis. All authors contributed toward the writing of the manuscript and approved the final submitted version.

## MELOR Investigators

Saedah S, Faculty of Education, University of Malaya, Tey NP, Faculty of Economics, University of Malaya, Siti Zawiah, MD, Center for Product Design and Manufacturing, Faculty of Engineering, University of Malaya, Noor Rosly H, Faculty of Built Environment, University of Malaya, Azriyati WNWAA, Faculty of Built Environment, University of Malaya, Ainoriza MA, Faculty of Built Environment, University of Malaya, Chan CS, Faculty of Computer Sciences and Information Technology, University of Malaya, Wee MC, Faculty of Computer Sciences and Information Technology, University of Malaya, Por LY, Faculty of Computer Sciences and Information Technology, University of Malaya, Zaharah H, Faculty of Education, University of Malaya, Norlida A, Faculty of Education, University of Malaya, Firdaus A, Department of Media Studies, Faculty of Arts and Social Sciences, University of Malaya, Siti Zaherah J, Faculty of Law, University of Malaya, Fatimah I, Center for Innovation in Medical Engineering (CIME) and Department of Biomedical Engineering, Faculty of Engineering, University of Malaya, Mas Sahidayana Mokhtar, Center for Innovation in Medical Engineering (CIME) and Department of Biomedical Engineering, Faculty of Engineering, University of Malaya, Morris T, School of Sport and Exercise Science and Institute of Sport, Exercise and Active Living, (ISEAL), Victoria University, Melbourne, Australia.

## Conflict of Interest

The authors declare that the research was conducted in the absence of any commercial or financial relationships that could be construed as a potential conflict of interest.
